# Prison Administration and the Training of Officials.—III

**Published:** 1898-04-09

**Authors:** 


### Modern Sociology.

### PRISON ADMINISTRATION AND THE TRAINING OF OFFICIALS.—III.

When describing the training of prison warders we mentioned our strong opinion, that such training ought to be made the invariable rule for all persons, male or female, who are allowed to take service under the Prison Commissioners. We have already adduced various reasons for our view that the educationary process should be absolutely universal, but there is a risk connected with the fact that at present only a favoured few obtain this needful preparation for their duties, which is so clearly stated by the secretary of the Howard Association in his letter to the *Times*, that we may as well quote his words: "The Commissioners and the two governors" (of the training schools at Chelmsford and Hull) "are alike particularly anxious that on leaving their training classes and entering on their future duties at other prisons, the probationers shall not at all pose as 'superior persons,' or set up Chelmsford and Hull as models elsewhere. On the contrary, it is assiduously impressed upon them that they should cultivate a modest demeanour and gratefully welcome the instructions of the governors and chief warders of the prisons amongst which they may be distributed, and that they shall willingly avail themselves of the experience of these and other older exemplars. And there is need for such a caution—for, in visiting a number of prisons lately I have found, in conversing with the officers, at least a little apprehension as to these new probationers, and of their training as if they were likely to 'come it over' their comrades in other gaols. But this is the very reverse of the wishes of the authorities. It is earnestly hoped it may be found that the Chelmsford and Hull training has not only given its subjects a valuable preliminary practice in actual prison duties under experienced seniors, but also that the counsels there given have tended to render the probationers more instead of less amenable to authority and discipline elsewhere, and also more ready than might otherwise have been the case to fraternise with new comrades in duty." Were the training universal this risk would, of course, be completely avoided; but we would go further, and since it is undoubtedly the desire of the authorities to make prison administration as perfect as possible, we would hope to see the day when, in addition to the regular general education of all intending warders, there shall be a selection made, both of men and women, who should be specially instructed in the skilled nursing of the sick and also of the insane. There are, of course, infirmaries in all prisons—two at least in each, on the male and female sides. These are served by warders—temporary assistants being often called in especially for night duty—but when the supply of attendants is not sufficient, as sometimes happens when there is an unusual amount of illness in the prison, it is frequently the custom, especially in the provincial gaols, to make use of such prisoners as seem most capable of being trusted. To this plan there are numerous and strong objections, but as matters are it is certainly very difficult to avoid it. When a convicted criminal, or one waiting trial, shows signs of aberration of mind, has attempted suicide, or is subject to fits, it is rightly and beneficently the rule that he or she should be ordered to be "under observation," as the official phrase is—*i.e.*, never to be left alone for a single moment, and watched night and day by some competent person. Such an order, of course, falls heavily on the regular staff, and is incompatible with their ordinary and imperative duties. It is in such a case that prisoners are sometimes employed. If a male patient is violent often two or three men have to be told off to restrain him, or otherwise minister to him, and such precautions, carried out by female warders, are often necessary among the women. A warder is always within call, and visits the patients and the guards continually, while the governor and the doctor do not fail to see them at stated intervals; but all this does not obviate the evils of the plan. The inevitable intercourse between the ailing patients and their nurses is highly undesirable, and is a fruitful source of serious and untoward consequences. It is much to be wished that every prison should have a supernumerary staff of trained warder nurses to be employed in all cases of sickness, so that it should never be necessary to have recourse to the prisoners for assistance. Doubtless such a plan would involve considerable additional expense, but we believe that the beneficial results would amply repay the outlay. The cases of attempted suicide which so frequently make their way into our English prisons—inasmuch as self-murder is a criminal act, as much as any other form of homicidal violence—are amongst the most trying and difficult from every point of view to all those whose duties, whether official or voluntary, bring them into close contact with prisoners. The conventional verdict, almost invariably brought in by coroners' juries on such cases when death has resulted, "Suicide under temporary insanity," is, as a rule, entirely untrue in the instances of attempted self-destruction which bring persons into the grasp of the law and consequent imprisonment. Generally speaking, while they listen quietly and silently to the chaplain's exhortations delivered, at least as regards female prisoners, the most frequent offenders in this respect, in the presence of an officer, they are very willing to express their real feelings when they have an opportunity of speaking to a prison visitor alone. Then quite calmly and rationally they state that life has become unendurable to them, they have exercised what they hold to be their independent right to put an end to it, and they hope to be more successful when they can again have their actions under their own control. It need hardly be said in these pages what is doubtless well known to our readers, that one result of the widespread predominance among the lower classes of Socialistic and Atheistic principles, has been to destroy utterly in their minds the idea that self-destruction is in any sense of the word a crime. Their life is given them; it is their one secure individual possession, and they deny to any Power the right to interfere with their disposal of it. This view of the case involves a somewhat serious problem when a prisoner, having undergone the prescribed term of his sentence, is of necessity liberated with the matter-of-fact certainty that he will at once use his freedom in making a more successful attempt on his own life. We believe that there might be found a remedy for this undesirable anomaly, and it leads us to certain forms of prison training which we are especially desirous to ventilate fully.

